# Risk Factors for Adult Depression: Adverse Childhood Experiences and Personality Functioning

**DOI:** 10.3389/fpsyg.2020.594698

**Published:** 2020-12-09

**Authors:** Paula Dagnino, María José Ugarte, Felipe Morales, Sofia González, Daniela Saralegui, Johannes C. Ehrenthal

**Affiliations:** ^1^Faculty of Psychology, Alberto Hurtado University, Santiago, Chile; ^2^Millennium Institute for the Study of Personality and Depression, Santiago, Chile; ^3^Center for Psychotherapy Research, Santiago, Chile; ^4^School of Psychology, Pontifical Catholic University of Chile, Santiago, Chile; ^5^School of Psychology, Universidad del Desarrollo, Santiago, Chile; ^6^Department of Psychology, University of Cologne, Cologne, Germany

**Keywords:** depression, impairments, risk factors, personality functioning, adverse childhood experiences

## Abstract

**Background:** Depressive disorder is one of the main health problems worldwide. Many risk factors have been associated with this pathology. However, while the association between risks factors and adult depression is well established, the mechanisms behind its impact remains poorly understood. A possible, yet untested explanation is the mediating impact of levels of personality functioning, i.e., impairments with regard to self and interpersonal.

**Method:** Around 162 patients were assessed at the beginning of their therapy, with regard to risk factors, such as sociodemographic, physical, hereditary (Information Form), and adverse childhood experiences (ACE; CTQ). Depressive symptoms (Beck Depression Inventory, BDI) and personality functioning (OPD-SQ) were also measured. Associations between the related variables as well as other possible covariates were examined by means of zero-order correlations and bootstrapping-based mediation analysis.

**Results:** Of all the risk factors taken into account, level of education and physical illness were associated with depression. On the other hand, the most significant predictor of depressive symptomatology was ACE, and this relationship was mediated by personality functioning. This indicates that patients presenting adverse childhood experiences are more likely to develop deficiencies in personality functioning, which in turn increases their likelihood of developing depressive symptomatology.

**Conclusion:** These results reaffirm the importance of incorporating risk and vulnerability factors such as personality functioning in understanding depression.

## Introduction

Mood disorders, such as depression, can be disruptive to daily functioning and well-being (Malinowski et al., 2017). Depressive disorders are one of the most prominent health problems worldwide (Whiteford et al., 2013). According to predictions, by 2030 depression will be the leading cause of disability, with a prevalence rate of 7.5% (WHO, 2017). In Chile, it is estimated that 15.8% of inhabitants present depressive symptoms, while 6.2% can be diagnosed with a depressive disorder according to the criteria of the DSM-IV (Ministerio de Salud Chile (MINSAL), 2016). Among those, some do not recover or develop a chronic mental disease (van Randenborgh et al., 2012). At the same time, what is usually described as “depression” is a heterogeneous construct (Fried and Nesse, 2015), which does not only point toward different symptom profiles, but also to a thorough assessment of the impact of risk factors and vulnerabilities. This is of practical relevance, as different pathways to the development and course of depression may also interact with the selection of treatments that suits the patient best (De la Parra et al., 2017), and identify strategies for relapse prevention (Ingram and Price, 2010).

Existing literature supports the relationship between different risk factors and depression, emphasizing that these factors are not incorporated in classification manuals, and therefore, current clinical guidelines do not yet consider their etiology and expression as indications regarding their management (Van Praag, 2010; Vitriol et al., 2017).

There is a great diversity of studies on risk factors for depression (e.g., Weich et al., 2002; Chapman et al., 2004), that can be grouped into four broad dimensions: sociodemographic factors, physical disease factor, hereditary factor, and adverse childhood experiences (ACE) factor.

Sociodemographic factors relate to gender (Piccinelli and Wilkinson, 2000; Cole and Dendukuri, 2003), marital status (Blazer, 1994; Blatt, 2004; Yan et al., 2011), age (Cole and Dendukuri, 2003; Kessler et al., 2005), educational level (Cole and Dendukuri, 2003; Kessler et al., 2003; Cacioppo et al., 2006; Akhtar-Danesh and Landeen, 2007; Bagana, 2013), and unemployment (Price et al., 2002).

Regarding physical illness, there are effects of chronicity (Egede, 2007; Martínez et al., 2007; Ehrenthal et al., 2016) and length (Knight et al., 2015) on depression, and that relates to a wide variety of diseases (e.g., Dickens et al., 2002; Evrard et al., 2010; Knight et al., 2015; Yu et al., 2016; Nerurkar et al., 2018; Nikendei et al., 2018), with complex bidirectional relationships.

With respect to genetic/hereditary factors, direct relatives of subjects suffering major depressive disorder have a significantly greater probability of developing a depressive disorder than the general population (Hodgson and McGuffin, 2012). The heritability of depression is estimated between 33 and 45%, being greater for some subtypes of depression (Sullivan et al., 2000; Silva, 2008). Regarding the mechanisms underlying this relationship, no specific genes involved have been found. Instead, many genes that have little relevance by themselves have been recognized, which explain a very small percentage of the total genetic component (Silva, 2002; Mitjans and Arias, 2012).

Finally, there is evidence that the occurrence of depression in adulthood is related to situations of emotional abuse, sexual, and physical neglect during childhood (Martins et al., 2019). ACE has important health consequences throughout the life cycle (Gilbert et al., 2009; Li et al., 2016). They are associated with multiple mental and biomedical problems, such as somatization, anxiety, hostility, fear, rejection, depression, distrust, substance abuse, obesity, hypertension, diabetes, cardiovascular problems, smoking, and sexual promiscuity (e.g., Comijs et al., 2013; Pajer et al., 2014; Adams et al., 2018). The presence of this experiences at an earlier age relates to greater chronicity and recurrence, comorbidity with anxiety and substance abuse, frequency of psychotic symptoms, interpersonal difficulty, higher suicidality, and poor response to pharmacological treatments (White, 2011; Hovens et al., 2012; Tunnard et al., 2014; Cantón and Cortés, 2015; Vitriol et al., 2017). But, the main focus in ACE has been on sexual abuse alone (e.g., Silk et al., 1995) or combined with physical abuse (e.g., Goldman et al., 1992).

Depression can be a very complex phenomenon. Risk factors are important to comprehend the onset, recurrence and prevalence of depression, but depression is a very heterogeneous syndrome (see e.g., de Vos et al., 2015; Fried and Nesse, 2015; Dagnino et al., 2017; Rantala et al., 2018) that must be accounted for having a personalized or tailored made treatment. In general terms, complex depression refers to the interaction between depression and personality, since more symptoms, like suicide or drug abuse will have a connection with the presence of personality disorders correlating with greater severity of depression (Klein et al., 2011; Clarkin et al., 2019). Comorbidity with personality disorders doubles the risk of negative outcomes with respect to diagnosis, prognosis and treatment, compared to depression without comorbidity with personality disorders (Grilo et al., 2004; Newton et al., 2006).

On the other hand, another alternative view using the Big Five personality model (Goldberg, 1993) has been found that higher neuroticism, less extraversion, and less awareness is significantly associated with depression, hopelessness, and suicidal ideation (Kotov et al., 2010; Koorevaar et al., 2013). These associations are consistent with the hypothesis that emotional instability and maladjustment play an important role in the development of negative affectivity such as depression (Chioqueta and Stiles, 2005).

Finally, other studies report that depressive styles such as self-critical and dependent (Blatt, 2004) will develop predispositions to stressors and show different responses for treatment (Dagnino et al., 2018), as well as personality vulnerabilities that are specific to each type of depressive style (OPD Working Group, 2008; Dagnino et al., 2017; De la Parra et al., 2017).

In addition to general personality, recent developments in the assessment of personality disorders in DSM-5 and ICD-11 point toward the importance of a careful consideration of dimensional models of personality functioning or integration (Bender et al., 2011; Tyrer et al., 2011, 2019; Zimmermann et al., 2019). This may be of special relevance for depression (Köhling et al., 2015).

The general concept of functioning is usually related to the concept of structure or organization of the personality. While structure or organization represents the theoretical concepts (the availability of capabilities), the concept of personality functioning refers to the observable manifestations of structural conditions (the actual use of capabilities; Dahlbender et al., 2006). Personality functioning evolves around two lifelong tasks, the development of capacities for interpersonal relatedness and the development of self-definition or identity, underpinned by functions oriented toward self-regulation and the relationship between the self and its internal and external objects.

Currently, there are a number of systems that measure personality functioning dimensionally. One of them, which have been in use in clinical and research settings for more than 15 years, is the Levels of Structural Integration Axis (LSIA) of the Operationalized Psychodynamic Diagnosis System (OPD-2; see Ehrenthal and Benecke, 2019). The OPD LSIA has a considerable amount of research with regard to reliability and validity (Zimmermann et al., 2012; Lorenzini et al., in evaluation). Moreover, it is associated with the Levels of Personality Functioning Scale (LPFS) of the DSM-5 Alternative Model for the Assessment of Personality Disorders (AMPD) on a conceptual (Zimmermann et al., 2012) as well as empirical level (Zimmermann et al., 2014). Personality functioning is not just associated with psychopathology such as depression (Ehrenthal et al., 2012; Zimmermann et al., 2012), but usually rooted in a history of early and repeated neglect and abuse. In fact Siegel (2001) refers to the “lower-mode” referring to a mode of processing ACE that impairs specific functions on personality, such as impulse control, capacity to maintain collaborative communication with others. Other researchers (e.g., Rogosch, Cicchetti, 2004; Brents et al., 2015; Granieri et al., 2018) have found that the exposure to multiple ACE associates with less flexible, and more maladaptive, personality patterns. Finally, the interrelationships between different types of childhood adversity and diverse personality dimensions have been reported by Schouw et al. (2020).

Therefore, personality functioning makes it an ideal candidate to explain a possible pathway between ACE and depression. Some level of impairment in personality functioning makes a substantial difference between patients with depression (Bach, 2018). Individuals with personality dysfunction are naturally more likely to experience depression, and less likely to remit, than the general population (Bender et al., 2011; Morey et al., 2013). However, to our best knowledge there are no studies that investigate this assumed mediation directly.

Considering this background, this study seeks to identify which risk factors are significantly related to depressive symptomatology, along with evaluating the influence that personality functioning has on this relationship. It is hypothesized that the effect of risk factors on depressive symptomatology will be mediated by personality functioning, which would develop with greater impoverishment if subjects have such risks and would thus result in greater depressive symptomatology.

## Materials and Methods

### Design and Participants

The study was based on a non-experimental, cross-sectional design. The sample consisted of 162 patients (72.33% women) aged between 18 and 70 (*M* = 31.55, *SD* = 11.27, detailed in Table 1). Around 66.67% of them presented higher educational level and the majority of them corresponded to a medium socioeconomic level. The average score obtained in the BDI Questionnaire (BDI-I-A; Beck et al., 1961) was 18.7 points (*DS* = 11.03).

**Table 1:** Risk factors and their association with depressive symptoms (Beck Depression Inventory, BDI).

Variables	*N*	*M*	*SD*	*F*	*df*	*p*	Cohen’s *d*
***Gender***							
Female	115	2.08	0.78	6.39	1.115	0.01	0.48
Male	44	1.66	0.88				
***Civil Status***							
Without partner	96	18.73	11.88	0.01	1.124	0.93	0.01
With partner	34	18.53	11.96				
***Educational Level***							
Lower educational level	43	21.54	11.44	4.08	1.124	0.05	0.36
High educational level	86	17.08	17.08				
***Actual physical disease***							
Yes	25	23.88	10.64	4.29	1.104	0.04	0.41
No	81	18.14	12.54				
***Genetic/hereditary factor***							
Yes	67	19.90	11.14	2.70	1.133	0.10	0.28
No	72	16.69	11.50				
***Adverse childhood experience***							
Yes	47	23.38	12.17	13.65	1.150	<0.001	0.60
No	113	16.35	10.20				

### Procedure

The consultants who agreed to participate in the study signed an Informed Consent Letter in which the ethical safeguards were pointed out, assuring confidentiality and voluntariness (the study was approved by the Alberto Hurtado University and the Gabriela Mistral University ethics committees). The sampling was done intentionally and for convenience among users who attended their first psychological consultation in private ambulatory psychological centers located in the Metropolitan Region of Chile (Psychological Treatment Center of Universidad Gabriela Mistral and Adult Psychotherapy Unit, Pontificia Universidad Católica de Chile). Inclusion criteria were a diagnosis of a depressive disorder by an experienced clinician according to the criteria of the ICD-10. The diagnosis was cross-validated by a standardized self-report questionnaire (see below).

The exclusion criteria were: under 18 years of age, consulting for primary substance abuse, psychotic symptoms, cognitive dysfunction, or eating disorder. The patients answered the Beck Depressive Inventory (BDI-I-A; Beck et al., 1961) before beginning their first session and those who scored higher than 13 points (Chilean cut off score, Valdés et al., 2017) entered the study. They then answered a Sociodemographic and Background Information Form and the OPD Structure Questionnaire (OPD-SQ; Ehrenthal et al., 2012).

### Instruments

#### Beck Depression Inventory (BDI-IA)

This self-report questionnaire evaluates depressive symptomatology (Beck et al., 1961) and is widely used throughout the world and in Chilean primary care settings (Alvarado et al., 2005). It consists of 21 items, which are scored on a scale from 0 to 3 (the higher the score, the greater the presence of depressive symptoms). The original version of the BDI-IA has adequate psychometric properties (Beck et al., 1988). Studies on the Chilean version show good internal consistency (*α* = 0.93), the ability to distinguish between depressive and non-depressive individuals, with a cut-off score around 13/14 points (Valdés et al., 2017).

#### Sociodemographic and Background Information Form

This form has been used in several studies of the Institute for the Study of Depression and Personality (MIDAP, Chile) and comprises a series of questions on patient data, including:

1. Sociodemographic factors (age, marital status, and educational level).
2. Physical disease factors (presence or absence of current physical illness).
3. Genetic/hereditary factor (presence or absence of previous or actual psychological problems in the family).
4. Adverse Childhood Experiences. They were assessed by a positive answer to at least one of two questions on physical and sexual abuse that were included in the Sociodemographic and Background Information. For physical abuse the question was: “When you were a child or teenager, did anyone living in your home hit you repeatedly with an object (belt, stick, etc.) or punched, kicked, or burned you as punishment?” For sexual abuse the question was “Can you recall any disturbing sexual experience with an adult before the age of 17, either a relative or someone in a position of authority, for example, a teacher?” To consider ACE, any of the questions must have a positive answer.

#### Personality Functioning (OPD-SQ)

Personality functioning was assessed with the OPD Structure Questionnaire (OPD-SQ). The OPD-SQ consists of 95 items (Ehrenthal et al., 2012) that assess eight personality dimensions. Each item is scored on a five-point Likert scale, from “I do not agree” to “I totally agree.” Higher scores imply lower levels of personality functioning. The mean of all the scales is an indicator of overall personality performance. Reliability studies in Germany with 1,110 subjects have shown Cronbach’s internal consistency scores between *α* = 0.71 and 0.91 for the subscales, and Cronbach’s α = 0.96 for overall performance in different samples (Ehrenthal et al., 2012). The OPD-SQ has been used in studies on for example, psychotherapy (Dinger et al., 2019), medical students’ stress (Bugaj et al., 2016), but also diabetes (Ehrenthal et al., 2019). It is significantly associated with overall symptom severity and the diagnosis of a personality disorder (Ehrenthal et al., 2012), but also other measures of personality difficulties (Zimmermann et al., 2014; König et al., 2016). Even more importantly, it correlates with OPD personality functioning expert ratings (*r* = 0.62; Dinger et al., 2019), and its 12-item short version (Ehrenthal et al., 2015) also with DSM-5 LPFS interview ratings (*r* = 0.78; Zettl et al., 2019). Initial adaptation and validation of the instrument for Chile (De la Parra et al., 2018) shows from an acceptable to high internal consistency scores (*α* = 0.71–0.93 for subscales). The Chilean validation (Lorenzini et al., in evaluation) showed an excellent internal consistency (*α* = 0.97) for the full sample. The clinical sample showed an *α* = 0.97, while the healthy control sample yielded an *α* = 0.93, it also showed good test-retest reliability (rho = 0.87; *p* < 0.001) and, discriminates between clinical and healthy samples.

### Data Analysis

The data analysis was carried out in R v.3.5.1 (R Core Team, 2018). As a first step, the descriptive statistics and the sample distribution of the variables were checked. Depressive symptomatology and OPD score were not normally distributed according to the Shapiro-Willks test (*W* = 0.97, *p* = 0.003; *W* = 0.97, *p* = 0.004). However, according to quantile-quantile plots and the levels of asymmetry in BDI (0.48) and in OPD-SQ (0.01), the violation of this assumption was not severe, in consequence, we proceeded to perform parametric association analysis such as Pearson correlation and ANOVA as both are relatively robust to the violation of the univariate normality assumption (Field et al., 2012). The results are presented in Tables 1, 2. The choice regarding which analysis (Pearson’s correlation or ANOVA) was decided based on the measurement level of each variable.

**Table 2:** Descriptive statistics and correlations (continuous variables).

Variables	*N*	*M*	*SD*	1	2	3
1. Age	142	31.51	11.27	-		
2. BDI	154	18.71	11.03	−0.59	-	
3. OPD-SQ	118	1.96	0.82	−0.17	0.71^***^	-

*** *p* < 0.001.

BDI, Beck Depression Inventory; OPD-SQ, Personality functioning according to the OPD Structure Questionnaire.

Later, based on theoretical considerations and the results obtained in the descriptive phase of the analysis, we then tested the mediational role of personality functioning on the association between ACE and depressive symptomatology. In addition, educational level and current physical illness were included as control variables, since they showed to have significant effects on depressive symptomatology. The mediation model was estimated with the “lavaan” package (Rosseel, 2012) in R, using a Robust Estimator of Maximum Likelihood (MLR) which gives more reliable results in samples with slightly skewed distributions (Beaujean, 2014), also, we used the Full Information Maximum Likelihood algorithm to treat the missing data in the endogenous variables of our model.

## Results

### Association of Risk Factors, Personality Functioning, and Depressive Symptomatology

As shown in Table 1, according to the ANOVA, women show higher levels of depressive symptoms than men. Also, depressive symptomatology is associated with educational level with a medium effect size, showing that people with higher levels of education tend to show fewer depressive symptoms. Similarly, the actual physical disease factor is associated with depressive symptomatology with a moderate effect size, meaning that individuals with a somatic disease had higher levels depressive symptoms. Finally, the existence of ACE was associated with depressive symptomatology with a moderate to large effect, showing that people who had ACE report higher levels of depressive symptomatology than those subjects who did not have this type of experiences. The other risk factors did not reach statistical significance (*p* > 0.05), also they showed small effect sizes (*d* < 0.30).

Correlation analyses (Table 2) indicated that personality functioning (OPD-SQ) is positively and strongly related with depressive symptomatology, i.e., less integrated personality functioning more presence of depressive symptomatology. When exploring the relationships between personality functioning and risk factors (see Table 3) according to the ANOVA, only ACE were associated with personality functioning, with a moderate to large effect size. Individuals who suffered some type of physical, and/or sexual abuse had lower levels of personality functioning. One factor that barely missed a conventional significance level was marital status, indicating that people without a partner tended to have lower levels of personality functioning than those who had a partner.

**Table 3:** Risk factors associated to personality functioning (OPD-SQ).

Variable	*N*	*M*	*SD*	*F*	*df*	*p*	Cohen’s *d*
***Civil status***							
Without partner	96	2.05	0.77	3.67	1.110	0.06	0.36
With partner	34	1.73	0.89				
***Educational level***							
Lower educational level	43	2.09	0.80	1.32	1.108	0.25	0.22
High educational level	86	1.90	0.81				
***Actual physical disease***							
Yes	25	2.14	0.74	0.81	1.101	0.37	0.18
No	81	1.98	0.83				
***Genetic/hereditary factor***							
Yes	67	2.00	0.80	0.68	1.111	0.41	0.16
No	72	1.88	0.81				
***Adverse childhood experience***							
Yes	47	2.25	0.75	10.64	1.114	0.002	0.62
No	113	1.76	0.82				

### Mediation Analyses

**Figure 1:**
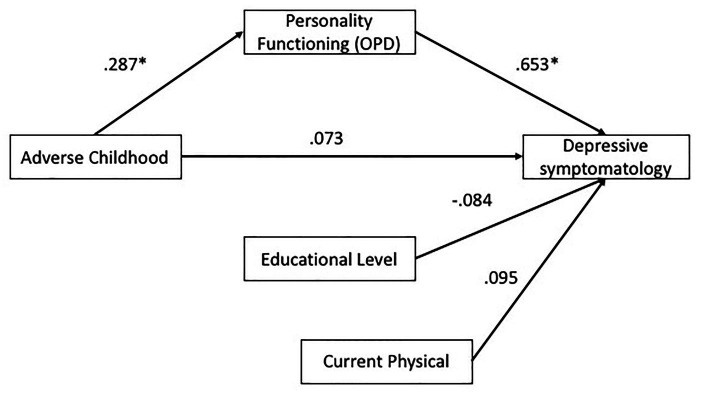
Mediation model of personality functioning on the relationship of adverse childhood experiences (ACE) and depressive symptomatology. ^*^p < 0.05, ^**^p < 0.01, ^***^p < 0.001.

In absolute terms, the model showed a good fit to data [X2(2) = 0.65, *p* = 0.72], in the same way the indicators of relative fit and parsimony show a good fit (CFI = 1, TLI = 1.07, RMSEA = 0.00), indicating that the proposed model fits properly with empirical data. The results of the mediation model are shown in Figure 1. ACE factors have no direct effect on depressive symptomatology any more in the presence of personality functioning (*B* = 0.073, *p* = 0.26). Based on this model, considering the variables studied, it can be observed that personality functioning is the most important predictor of depressive symptomatology (*B* = 0.653, *p* < 0.001). In addition, the indirect effect of ACE on depressive symptomatology through personality functioning was significant (*B* = 0.193, *p* = 0.004). These results indicate that people who had ACE (physical or sexual abuse) are more likely to have a less integrated personality functioning, and consequently, this increases their likelihood of developing depressive symptomatology later in life.

## Discussion and Conclusion

The present study explored the relationship between risk factors for depression, personality functioning, and depressive symptomatology. One of the first results was that impairments on personality functioning were associated with a greater presence of depressive symptoms, being consistent with previous studies (Bender et al., 2011; Ehrenthal et al., 2012; Zimmermann et al., 2012; Morey et al., 2013; Dagnino et al., 2017).

In relation to risk factors, the results show several associations. First, it was found that patients with higher levels of education tend to show less depressive symptomatology. There are previous studies that assess the relationship between socioeconomic and educational level on mental health, specially depression (Eaton et al., 2001; Lorant et al., 2003). This may have to do with poorer coping styles, ongoing life events, higher stress exposure, and weaker social support on people with lower educational level. All of which leads to a difficult life quality with a lower quantity and/or access to resources to face life (Cacioppo et al., 2006; Bagana, 2013).

On the other hand, it was found that the presence of physical illnesses increases the probability of suffering depressive symptoms. There are several studies that support this relationship, however focused especially on chronic diseases (Egede, 2007; Martínez et al., 2007). What is found is that in the case of the present study, the question about diseases was broad (presence/absence), so it was ascribed to the subjectivity of the patient to consider whether he/she had a physical disease. It could be hypothesized that having more depressive symptoms makes subjects more receptive to feeling “sick,” so it is important that in the future this measure can specify what type of disease is afflicting the patient and as refers Chen et al. (2020) the illness perception of the patient. Depression and medical illness can be explained in several ways, for example, people suffering for a medical issue may have limitations in their daily life (e.g., pain, diabetes, cefalea, etc.), which can lead to social isolation or physical innactivitue, smoking, poor diet, all of which can be considered as risk factors for depressive symptomatology (Rosenblat et al., 2020).

Although theoretical-empirical background supports a relationship between marital status and depression (e.g., Yan et al., 2011), the present study did not show this result. This may be due to sample size, which influenced the effect of having a partner on personality functioning and depressive symptomatology not appearing, despite having an important effect size. In addition, gender could not be considered in the analysis, given the high percentage of women in the sample (also a risk factor supported by the literature).

The results show that patients with ACE report higher levels of depressive symptomatology, and these experiences become the only risk factor with a significant ability to predict the presence of depressive symptoms. Studies such as Chapman et al. (2004) confirm that experiencing multiple forms of domestic abuse or dysfunction during childhood can have particularly detrimental consequences for the mental health of adults. Furthermore, these authors suggest that detection of an ACE should alert professionals to assess the patient for a history of exposure to other forms of abuse or domestic dysfunction.

A second important goal of this study was to estimate the mediating effect of the personality functioning in the relationship between depressive symptomatology and risk factors, finding a significant mediating effect, which entails important implications for understanding the causal mechanism that ACE have on depression. The results speak of a continuous process, in which the adverse experience in childhood deteriorates personality functioning, which in turn increases the probability of depressive symptomatology. This is of great relevance, since there is abundant literature on ACEs as risk factors for depression (e.g., Martins et al., 2011; Gibb et al., 2013; Tunnard et al., 2014), but not so with respect to how this relationship works or what processes intervene in it. ACEs can be understood as experiences of acute stress, which has been considered as highly relevant in the stress-diathesis model of psychiatric disorders. This model goes in line with the results of this study since it focuses on the relationship between stress and its response, which is considered as mediated by personality characteristics (Slavik and Croake, 2006).

In the same vein and taking up the distinction made by Ingram and Price (2010) between vulnerability and risk factors, we can consider that personality functioning shows to be a vulnerability factor, given that it is endogenous and relatively stable, requiring deeper and more causal mechanisms (Dobson and Dozois, 2008). On the other hand, ACE would be considered as the risk factor that predisposes to it. This may be key for clinical interventions, since there is greater probability that impairments as vulnerability (personality functioning) must be worked on initially or throughout the process, and not the risk factor itself. This is consistent with studies that relate depression and personality (Klein et al., 2011; Dagnino et al., 2017) and approaches that explain the relationship between these constructs from a “pathoplastic” explanation; proposing that personality would have an influence on how depression is expressed, in terms of severity and response to treatment, among others (Klein et al., 2011).

Considering the results, and the multiple dimensions through which it is possible to understand depression as a clinical and heterogeneous phenomenon (Van Praag, 2010; Fuller-Thomson et al., 2016; Milaneschi et al., 2016), the aim of the therapeutic work is to determine what it means to suffer depression for each patient in particular (with his/her requirements, strengths, and risks). Therefore, the role played by personality functioning in the relationship between depressive symptomatology and risk factors is fundamental for diagnosis, intervention, and prognosis.

From a clinical perspective, it is relevant to achieve knowledge both of the vulnerabilities that underlies symptomatology (in this case, personality functioning) and of the risk factors, especially the ACE. This, in order to be able to make an approach that not only addresses the current symptomatic manifestation of the patient, but also takes a complete look at its complexity and heterogeneity, including interventions that are conducive to the patient’s profile, enriching an idiosyncratic and patient-centered treatment (Van Praag, 1989; Gabbard and Simonsen, 2007; Dagnino et al., 2017).

Nevertheless, the results of this study must be considered with some limitations. First, regarding sample composition, a great majority of this research sample had a higher educational level, meaning that the analyses carried out with this variable could be biased, as it is not representative for other educational levels. Also, in relation to the variable “gender,” the sample presented a significantly greater proportion of women than men, which could have influenced the absence of a relationship between this variable and depression; a relationship that is widely supported by existing literature (Piccinelli and Wilkinson, 2000; Cole and Dendukuri, 2003).

When considering ACE there are several limitations. Firstly, it was measured through only two questions and not using self-reported instruments such as CTQ or MACE. Secondly, physical and/or sexual abuse where the only ACE considered, and some studies have shown that emotional abuse is a significant risk factor involved in the pathogenesis of depression (Martins et al., 2019). In considering these limitations, the importance of adverse experiences must be accounted, such as the physical and/or sexual abuse, which must be investigated in the future thoroughly. Continuing with ACE measures, a third limitation was temporal, since the measure was retrospective, being susceptible to memory biases.

In the future, it would be relevant to delve into which specific aspects of personality functioning predict depression and how this relationship works. Also, it would be important to open a discussion regarding other mechanisms by which depressive symptomatology are increased by risk factors. In other words, besides personality functioning, what other factors could mediate these relationships? This applies with greater force to the variable schooling and physical illness, which have important effect sizes.

## Data Availability Statement

The datasets presented in this article are not readily available due to confidentiality issues. Requests to access the datasets should be directed to pdagnino@uahurtado.cl.

## Ethics Statement

The studies involving human participants were reviewed and approved by Alberto Hurtado University Ethics Committee (Santiago, Chile) and Gabriela Mistral University Ethics Commmittee (Santiago, Chile). The patients/participants provided their written informed consent to participate in this study.

## Author Contributions

All authors contributed to the article and approved the submitted version.

### Conflict of Interest

The authors declare that the research was conducted in the absence of any commercial or financial relationships that could be construed as a potential conflict of interest.
